# Management of Breast Intraductal Papilloma Diagnosed on Core Needle Biopsy: Excision or Follow-up?

**DOI:** 10.7759/cureus.54716

**Published:** 2024-02-22

**Authors:** Mishal Gillani, Romana Idress, Shaista Afzal, Maria Khan, Hania Shahzad, Abida K Sattar

**Affiliations:** 1 Department of Medicine, Aga Khan University Hospital, Karachi, PAK; 2 Department of Histopathology, Aga Khan University Hospital, Karachi, PAK; 3 Department of Radiology, Aga Khan University Hospital, Karachi, PAK; 4 Department of Surgery, Aga Khan University Hospital, Karachi, PAK

**Keywords:** excision surgery, breast cancer outcomes, atypia, core needle biopsy, intraductal papilloma

## Abstract

Introduction

Management of intraductal papillomas (IDPs) diagnosed on core needle biopsy (CNB) remains controversial. We report our experience of IDPs identified on CNB, our institutional rates of upgradation to atypia/malignancy as well as radiologic/pathologic features that may allow selection for surgery as well as those for safe observation.

Methods

The study is a retrospective review of patient records from 2012 to 2019, at a tertiary care hospital in Pakistan. Data was analyzed using Statistical Package for Social Sciences (SPSS), version 21.0 (IBM Corp., Armonk, NY). Associations between various patient factors were assessed using Pearson’s chi-square test.

Results

This study included a total of 55 female patients with IDPs, with a mean age of 54.67 ± 15.57 years. On CNB, 69.1% (n = 38) of patients had IDP without atypia while 30.9% (n = 17) had IDP with atypia, with single IDPs being the most common lesions on excisional biopsy. Overall, of all CNB-diagnosed IDPs, only 4/55 (7.3%) demonstrated upgradation (3/4 to DCIS, 1/4 showed atypia) on excisional biopsy, and all these upgraded cases had failed to demonstrate atypia on initial CNB.

Conclusion

CNB-identified cases of IDPs are rarely upgraded on excision and thus routine excision in all cases may be unnecessary. Appropriate patient selection based on radiology-pathology findings should be done. Those with suspicious findings on imaging as well as those that demonstrate atypia on CNB must be excised.

## Introduction

Intraductal papillomas (IDPs) are tumors composed of epithelial cells with a fibrovascular core that account for approximately 5% of all biopsied breast tumors [1]. Certain clinical, radiologic, and pathologic characteristics of benign IDPs may indicate an increased risk of malignancy, such as there being multiple IDPs [2], atypia on core needle biopsy (CNB) [3], and the papilloma estimated to be >1 cm on ultrasound [4]. Papillomas on excision may be upgraded to malignancy, with upgradation rates of 0 to 20% [5-7].

Management of IDPs diagnosed with CNB remains controversial. While many suggest routine excision of all lesions to rule out co-existing malignancy [8,9], some suggest selective surgical excision of IDPs with atypia only, on CNB [10] and others again suggest observation [11-14]. Upgradation upon excision may be dependent on many factors and institutions may need to assess their own upgradation rates to develop institutional guidelines [8,14].

We report our experience with female patients diagnosed with breast IDPs identified on CNB. Our report includes institutional rates of upgradation to atypia/malignancy, along with radiologic/pathologic features. These features may guide the selection for surgery and identify criteria for safe observation.

## Materials and methods

Study setting and population

The protocol of this retrospective study was exempted by the institutional review board (No. 2020-4917-11362) of Aga Khan University Hospital (AKUH). A prospectively maintained institutional pathology database at the AKUH was retrospectively reviewed to identify all CNB-diagnosed cases of IDPs between January 1, 2012, to December 31, 2019. All cases of IDPs that underwent surgical excision were included in our analysis. In contrast, cases with incomplete data or those that did not have surgical excision were systematically excluded from the study. All CNBs were reviewed again by study pathologists and radiologic-pathologic (Rad-Path) correlation was performed by study radiologists.

Statistical analysis

Data was analyzed using Statistical Package for Social Sciences (SPSS), version 21.0 (IBM Corp., Armonk, NY). Categorical variables have been reported as proportions. Associations between various patient factors and CNB results have been assessed using Pearson’s chi-square test. A p-value of <0.05 is considered statistically significant. 

## Results

**Figure 1:**
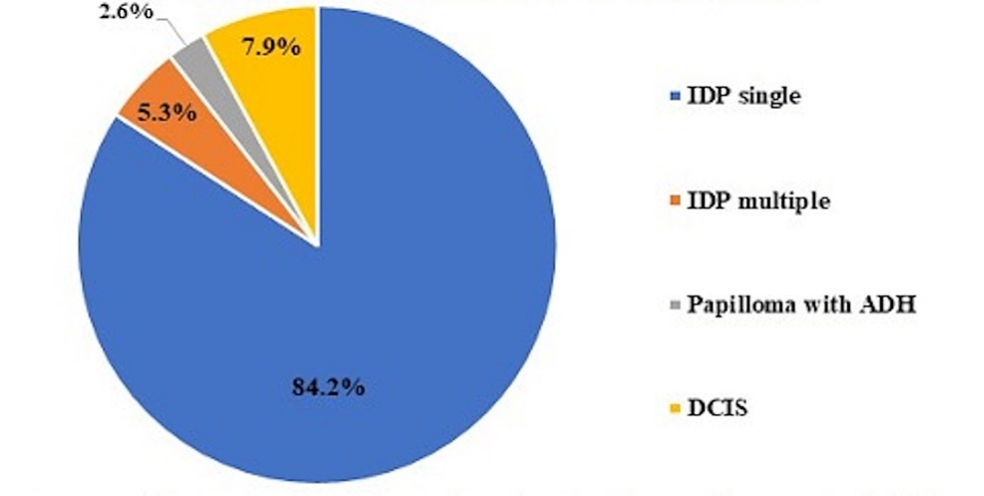
Pathology on surgical excision of CNB-proven IDPs without atypia ADH: atypical ductal hyperplasia; CNB: core needle biopsy; DCIS: ductal carcinoma in situ; IDP: intraductal papilloma

A total of 55 female patients with breast IDPs underwent surgical excision and were included for review. The mean age was 54.67 ± 15.57 years. On CNB, 38/55 (69.1%) had IDP without atypia while 17/55 (30.9%) had IDP with atypia. When cases of IDP without atypia diagnosed on CNB were excised, histopathology confirmed single IDPs in the majority (84.2%) of cases, while 2/38 (5.3%) showed multiple IDPs. Additionally, 1/38 (2.6%) had IDP with atypical ductal hyperplasia (ADH), and 3/38 (7.9%) were upgraded to ductal carcinoma in situ (DCIS) as shown in Figure 1.

**Figure 2:**
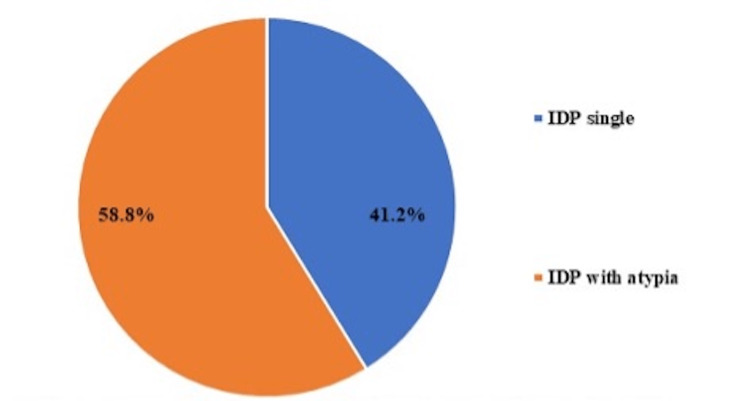
Pathology on surgical excision of CNB-proven IDPs with atypia CNB: core needle biopsy; IDP: intraductal papilloma

When CNB-diagnosed IDPs with atypia were excised, 7/17 (41.2%) demonstrated single IDP on excision without residual atypia or malignancy, while 10/17 (58.8%) had IDPs with residual atypia but without evidence of co-existing malignancy, as shown in Figure 2.

Overall, of all CNB-diagnosed IDPs, only 4/55 (7.3%) demonstrated upgradation (3/4 to DCIS, 1/4 showed atypia) on excisional biopsy, and all these upgraded cases had failed to demonstrate atypia on initial CNB. On Rad-Path correlation, of those that upgraded to DCIS or atypia on excision, 3/4 (75%) had a mass and/or micro-calcifications on mammography, while 1/4 did not have any significant findings on mammography or ultrasound (US) (p-value = 0.003).

## Discussion

To date, the focus of literature has been on the management of CNB-diagnosed cases of IDPs, with and without atypia [12,15]. Perhaps the most important aspect of research on IDPs is their rate of upgradation or upstaging to malignancy. It is through this rate that surgeons can categorize a lesion to be low or high-risk and thus determine the need for surgical excision [16] or safe follow-up, thus avoiding unnecessary surgical morbidity and expense. 

This study identified the upgradation rate of IDPs in our population and also identified the characteristics of IDPs in our setting. The upgradation rate in our study of 7.3% was much lower when compared to another study which reported a 45% upgradation rate [17]. Overall, upgradation rates have varied widely across literature, reaching up to 20% [5,6] and may be dependent on multiple factors of which the presence of atypia on CNB has been the strongest predictor of upgradation to malignancy with rates up to 45% [8,18], though none of our atypia cases had evidence of malignancy on surgical excision. Radiologic-pathologic correlation remains of paramount importance when deciding to proceed with surgery. Maxwell et al. suggested excision for IDPs associated with suspicious microcalcifications on mammography (as the upgradation rate of IDPs with microcalcifications was 6/29 (21%) compared to 5/67 (7%) for those without microcalcifications [19]. Similarly, Sakr et al. found microcalcifications (is this macro or micro?) to indicate a higher risk for malignancy [20]. Though Deshaies et al. did not find microcalcifications alone to be predictive of increased risk of malignancy, in our cases of CNB-diagnosed IDP with an associated mass and/or calcifications, the upgradation rate was high at 75% [21]. Race and ethnicity have not been demonstrated to impact upgradation or the decision to excise [22]. Most reported studies have limited sample sizes, ranging from 100 to 200 [12,16-18], with some studies of less than 100 [1].

There are limitations to this study, including the small sample size and reliance on single-institution data, which limit its inherent statistical power. Nevertheless, many statistically and clinically significant differences were observed. Though not well-documented in our own study, other factors that may impact the findings on surgical excision could be related to the needle gauge used to obtain cores, as well as the number of cores taken. In certain situations, pathology limited to a small area may be entirely removed in the process of percutaneous biopsy sampling, thus resulting in an even lower upgradation rate. Consequently, a more extensive, multicenter, prospective study that systematically considers variables such as imaging-based predictions, needle gauge, number of cores, and final histopathology following excision is warranted. Such an approach would enhance our understanding, offer more robust guidance for patient selection, and mitigate the risk of unnecessary surgical morbidity.

## Conclusions

It is rare for IDPs diagnosed on CNB to upgrade on excision; therefore, routine excision in all cases may be unnecessary. Appropriate patient selection of those who are likely to have malignancy or will be identified with atypia in order to offer appropriate treatment or prophylaxis is essential. For this, radiology-pathology correlation must be performed. Those with suspicious findings on imaging, such as microcalcifications and/or masses, as well as those that demonstrate atypia on CNB, should be considered for excision.
